# Accelerated Growth Rate and Increased Drought Stress Resilience of the Model Grass *Brachypodium distachyon* Colonized by *Bacillus subtilis* B26

**DOI:** 10.1371/journal.pone.0130456

**Published:** 2015-06-23

**Authors:** François Gagné-Bourque, Boris F. Mayer, Jean-Benoit Charron, Hojatollah Vali, Annick Bertrand, Suha Jabaji

**Affiliations:** 1 Department of Plant Science, Macdonald Campus of McGill University, 21,111 Lakeshore Rd. Ste-Anne-de-Bellevue, Québec, CANADA, H9X 3V9; 2 Facility of Electron Microscopy Research (FEMR) McGill University, 3640 University Street, Montréal, Québec, CANADA, H3A 0C7; 3 Soils and Crops Research Development Center, Agriculture and Agri-Food Canada, 2560 Hochelaga Boulevard, Québec City, Québec, CANADA, G1V 2J3; Chinese Academy of Sciences, CHINA

## Abstract

Plant growth-promoting bacteria (PGB) induce positive effects in plants, for instance, increased growth and reduced abiotic stresses susceptibility. The mechanisms by which these bacteria impact the host plant are numerous, diverse and often specific. Here, we studied the agronomical, molecular and biochemical effects of the endophytic PGB *Bacillus subtilis* B26 on the full life cycle of *Brachypodium distachyon* Bd21, an established model species for functional genomics in cereal crops and temperate grasses. Inoculation of *Brachypodium* with *B*. *subtilis* strain B26 increased root and shoot weights, accelerated growth rate and seed yield as compared to control plants. *B*. *subtilis* strain B26 efficiently colonized the plant and was recovered from roots, stems and blades as well as seeds of *Brachypodium*, indicating that the bacterium is able to migrate, spread systemically inside the plant, establish itself in the aerial plant tissues and organs, and is vertically transmitted to seeds. The presence of *B*. *subtilis* strain B26 in the seed led to systemic colonization of the next generation of *Brachypodium* plants. Inoculated *Brachypodium* seedlings and mature plants exposed to acute and chronic drought stress minimized the phenotypic effect of drought compared to plants not harbouring the bacterium. Protection from the inhibitory effects of drought by the bacterium was linked to upregulation of the drought-response genes, *DREB2B-like*, *DHN3-like* and *LEA-14-A-like* and modulation of the DNA methylation genes, *MET1B-like*, *CMT3-like* and *DRM2-like*, that regulate the process. Additionally, total soluble sugars and starch contents increased in stressed inoculated plants, a biochemical indication of drought tolerance. In conclusion, we show a single inoculation of *Brachypodium* with a PGB affected the whole growth cycle of the plant, accelerating its growth rates, shortening its vegetative period, and alleviating drought stress effects. These effects are relevant to grasses and cereal crops.

## Introduction

Plant-growth promoting bacteria (PGB) are mainly soil and rhizosphere-derived organisms that are able to colonize plant roots and positively influence plant growth or reduce disease [1]. Several strains of *Bacillus* species, representing typical PGB have been widely studied and applied as commercialized products for efficient control of disease [2]. *Bacillus* spp. stimulate plant growth, increase yield and reduce pathogen infection without conferring pathogenicity [1]. The proposed mechanisms for plant growth promotion include increased nutrient availability, synthesizing plant hormones and production of volatiles [3–5]. Considerable progress has been made in understanding the mechanisms underlying *Bacillus*-mediated tolerance to biotic stress [6–8] however, information on *Bacillus* strains mitigating abiotic stress symptoms is limited [9,10] and the mechanisms underlying abiotic tolerance are largely elusive because most of the studies focus on evaluating plant growth promoting effects [11].

It has been demonstrated that a range of bacterial endophytes, the majority of which are derived from the rhizosphere, colonize the plant’s interior and many of them have been reported to improve plant growth [12]. Following rhizosphere establishment, endophytes may colonize various plant organs [12–14]. *Bacillus* species, considered as root colonizing rhizosphere competent bacteria are often also found as colonizers of internal tissues of plants [14,15]. Reports on the endophytic colonization of *Bacillus subtilis* are few focusing on the internal colonization of roots [16,17] and leaves of young seedlings [18] grown for a short period of time. However, no reports exist in which internal colonization, establishment and spread of *B*. *subtilis* were followed in vegetative and reproductive plant growth stages.

We previously reported on a strain of *B*. *subtilis* B26, which was isolated from leaf blades and seeds of the bioenergy crop switchgrass (*Panicum virgatum* L.), and demonstrated that it is a growth enhancer of four-week-old switchgrass seedlings, as well as its ability to migrate from the roots to aerial parts of the seedlings [19], strongly suggesting that it behaves as a competent endophyte [12]. *B*. *subtilis* B26 culture filtrate contains several well-characterized lipopeptide toxins and phytohormones [19]. These qualities suggest that the endophytic ability of this strain is a biological requirement for survival in nature and has strong potential as bio-inoculant for biomass enhancement of bioenergy crops and boosting the plant’s defence against abiotic stress such as drought stress. In this study, we aim to investigate whether the internal colonization of *B*. *subtilis* endophytic strain B26 might modulate gene expression in plants, and the genes so expressed provide clues as to the effects of B26 in plants, and trigger the plant defence mechanisms to enhance resistance against abiotic stress.

Studies based on defined model systems with reduced complexity will be important in elucidating the molecular mechanisms underlying *B*. *subtilis*-mediated growth promoting abilities and the physiological changes enhancing their adaptation to abiotic stress. *Brachypodium distachyon* is a temperate monocotyledonous plant of the *Poaceae* grass family that is now established as the model species [20] for functional genomics in cereal crops and temperate grasses like switchgrass [21]. *Bachypodium* is an annual, self-fertile plant with a life cycle of less than 4 months and a small nutrient requirement throughout its growth [20]. Many mutant accession lines and genetic web base free tools are available. *Brachypodium* has proven particularly useful for comparative genomics and its utility as a functional model for traits in grasses including cell wall composition, yield, stress tolerance, cell wall biosynthesis, root growth, development, and plant-pathogen interactions had been recently reported [22,23]. Despite these advancements in the diverse utility of *Brachypodium*, the usefulness of *Brachypodium* to study plant-bacterial endophyte interactions has not yet been explored.

Here we report that a single inoculation of *Brachypodium distachyon* young seedlings with the strain *Bacillus subtilis* B26, exerts phenotypic effects throughout the whole life cycle of the plant leading to an acceleration of flowering, seed set times and senescence in inoculated plants. We also demonstrate that strain B26 colonizes intra- and intercellularly vegetative and reproductive tissues causing cellular structural changes. Moreover, in response to acute and chronic drought treatments, we show that *B*. *subtilis* B26 does not only modulate *Brachypodium* drought-responsive genes but also has an effect on the global DNA methylation and the genes that regulate the process. This study provides novel and interesting information about long-term effects of a PGB on plant development under normal and drought stress conditions contributing to the knowledge on these relevant biological interactions in grasses.

## Results

### Inoculation of *Bacillus subtilis* strain B26 improved production of biomass and seeds

**Fig 1:**
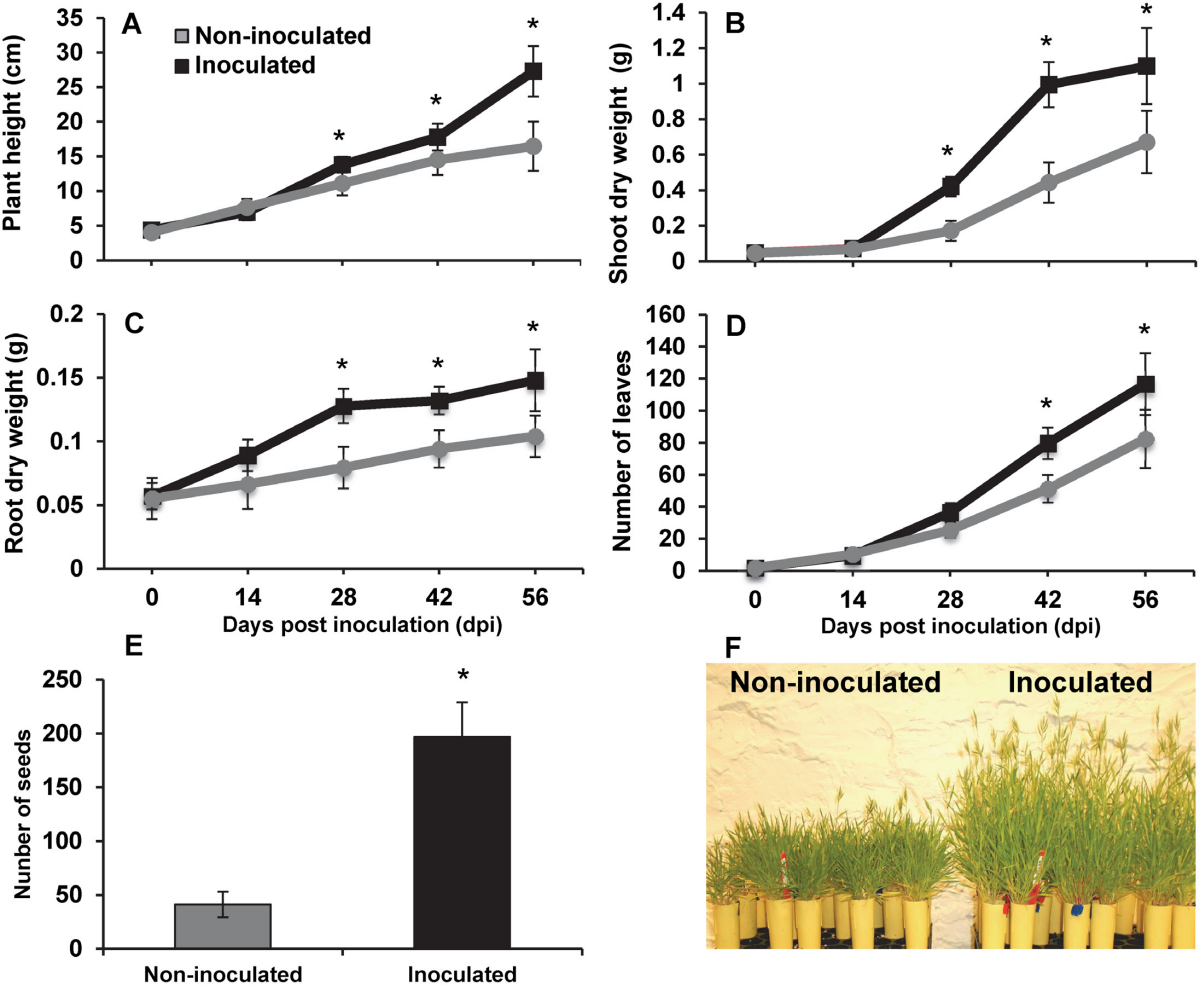
Phenotypic effects of the endophytic *bacteria Bacillus subtilis* B26 on the life cycle of *Brachypodium*. Effects of the inoculation of *Brachypodium* plants with strain B26 on: (A) plant height (B) shoot dry weight (C) root dry weight (D) number of leaves (E) number of seed, and (F) picture of non-inoculated and inoculated plants. * Represents a statistically significant difference.

The model plant *Brachypodium distachyon* provides many advantages for genomics in grasses. In this study, we sought to examine the ability of *B*. *subtilis* B26 to promote growth of *Brachypodium* in growth chamber experiments. Starting at 28 days post inoculation (dpi), inoculated *Brachypodium* plants showed a significant (*P* <0.05) and steady increase in plant height, shoot and root dry weights and number of leaves (Fig 1) when compared with control non-inoculated plants. At 56 dpi with *B*. *subtilis* B26, the BBCH97 stage (Table 1) [24] at which *Brachypodium* had seeds, significant growth promotion with a 65.8%, 63.79%, 42.29% and 41.50% increases in plant height (Fig 1A), shoot (Fig 1B) and root (Fig 1C) dry biomass and number of leaves (Fig 1D), respectively was observed, suggesting that *B*. *subtilis* B26 behaved as a PGPR on *Brachypodium* (Fig 1F). Additionally, there was a significant difference in seed production between inoculated and non-inoculated plants (Fig 1E). Inoculated plants produced 64% more seed heads than control plants (S1 Fig), indicating that more tillers became reproductive in inoculated plants. Notably, inoculated plants produced 121% more spikelets (S1 Fig) resulting in approximately 377% increase in seed yield (Fig 1E). Concentrations of N, P, K and Mg in above ground tissues of inoculated plants were significantly lower at 42 dpi (S2 Table), indicating that the growth promoting ability is not related to increase in nutrients.

**Table 1:** Scale for phonological growth stages in *Brachypodium distachyon*.

Dpi*	Stage**	Description
0	BBCH13	3rd true leaf unfolded
14	BBCH45	Late boot stage: flag leaf sheath swollen
28	BBCH55	Middle of heading: half of inflorescence emerged
42	BBCH77	Late milk
56	BBCH97	Plant dead and collapsing
70	BBCH99	Harvested seed

*Days post inoculation.

**Biologische Bundesantalt Bundessortenamt and CHemische industrie (BBCH) growth scale [24].

### Successful and stable colonization of vegetative and reproductive organs of *Brachypodium distachyon* by *B*. *subtilis* strain B26

**Fig 2:**
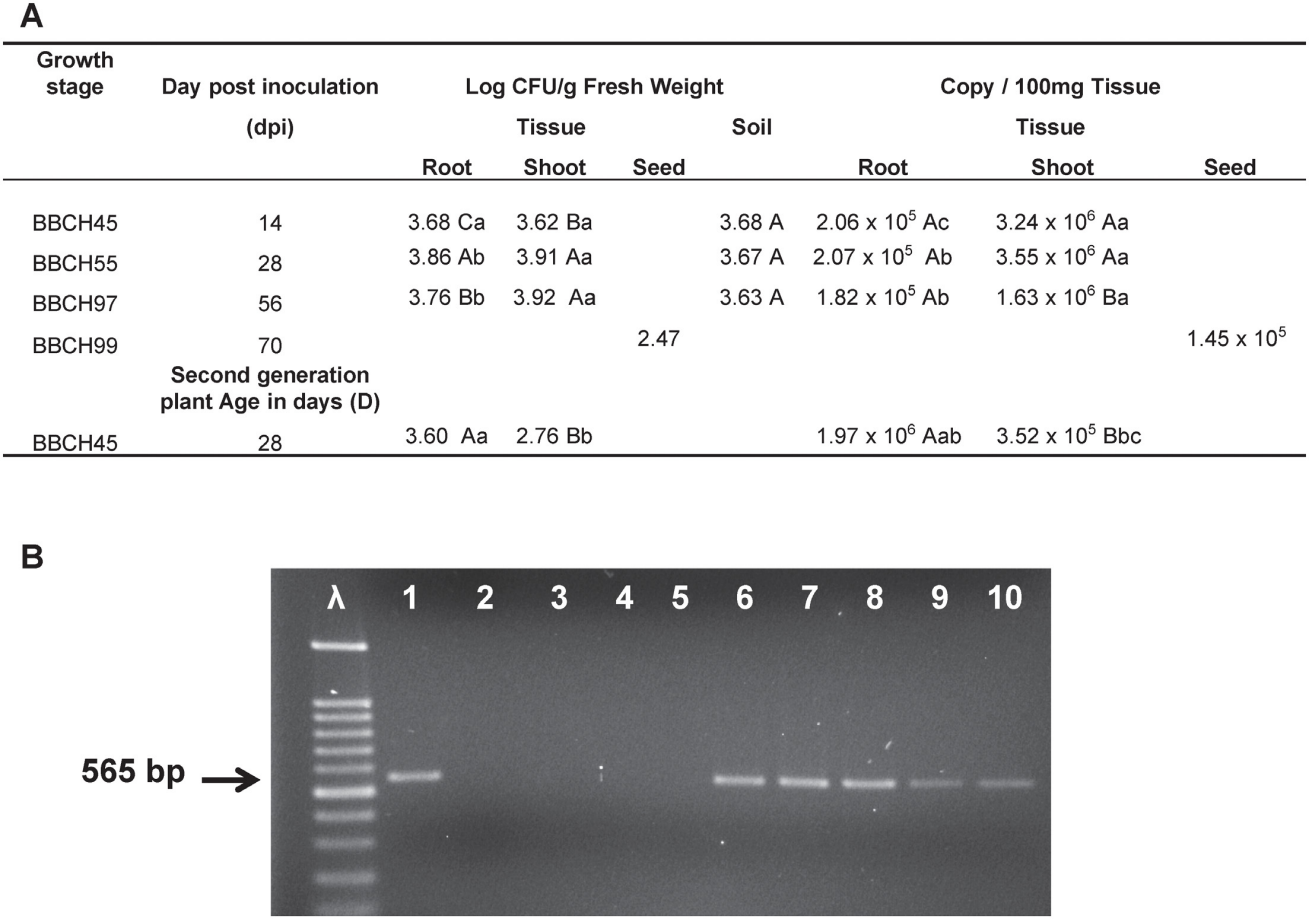
Dynamics of *B*. *subtilis* B26 in the host plant. (A) CFU and DNA copy number of *B*. *subtilis* B26 in roots, shoots, seeds and rhizosphere soil. Upper case letters represent differences in between time points of the same tissue/soil, and lower case letters represent differences between different tissues at the same time point. (B) PCR detection of *B*. *subtilis* B26 in different tissues using species-specific primers. Lane 1, pure *B*. *subtilis* B26 DNA; Lane 2, no template; Lanes 3 to 5, non-inoculated plant tissues of root, shoot and seed at D63; Lanes 6 to 8, inoculated plant tissues of root, shoot, seed at D63; Lanes 9 and 10, root and shoot tissues of second generation plant at D28.

The success of internal and systemic colonization of *Brachypodium distachyon* by *B*. *subtilis* B26 was confirmed by culture-dependent and independent methods. Re-isolation and quantification of *B*. *subtilis* strain B26 by the plating method in different surface-sterilized tissues of first and second generations of *Brachypodium* plants after soil drench treatment with *B*. *subtilis* clearly demonstrate that *B*. *subtilis* B26 can form sustaining endophytic populations in roots, shoots and seeds as well as in the soil around the roots of *Brachypodium* (Fig 2). Following rhizosphere colonization of *Brachypodium*, bacterial counts within root tissue changed with the plants growth stage, while numbers of CFUs in shoots stabilized over the last two growth stages (BBCH 55 and BBCH97; Table 1). However, population numbers in shoots were consistently higher than in roots indicating that there was successful translocation from roots to shoots. CFU numbers in rhizosphere soil remained stable over time. Moreover, vegetative tissues of the *Brachypodium* young plants (BBCH45) that originated from seeds of the first generation sustained similar population numbers to those from the first generation for the corresponding growth stage (Fig 2A). Population numbers in *Brachypodium* seeds were lower by a factor of 10 compared to other tissues. Rhizosphere soil and surface sterilized tissues of control plants did not yield cultivable bacterial colonies.

Additionally, the presence of *B*. *subtilis* B26 in different tissues of *Brachypodium* was confirmed by qPCR in inoculated plants (Fig 2A). An amplicon with the expected product size of 565 bp was successfully amplified using species-specific primers for *B*. *subtilis* B26 from DNA extracted from each tissue type (Fig 2B). Non-inoculated tissue samples tested negative for the presence of *B*. *subtilis* B26 (Fig 2B). Absolute quantification by qPCR of *B*. *subtilis* B26 copy numbers sustained the same numbers in the root at all growth stages and a small decrease in shoot tissue, with 10 times more copy in *Brachypodium* shoots compared to roots (Fig 2A). Copy numbers in seeds of *B*. *subtilis* B26 were the lowest of all tissues tested. Second generation plant tissue showed the highest concentration of endophyte in the root and a lower amount in the shoot than in the inoculated plants at corresponding growth stages.

To assess whether the systemic colonization of *Brachypodium distachyon* by *B*. *subtilis* B26 triggers an immune response, we monitored the transcript accumulation levels of the pathogenesis-related gene in inoculated and non-inoculated plants using qRT-PCR. Since the *PR1* gene is not fully characterized in the *Brachypodium* model, we first sought to determine if an exogenous application of salicylic acid (SA) could trigger a transcripts accumulation of the selected *Brachypodium PR1*-like gene (S2 Fig). As expected, *Brachypodium* plants sprayed with 5 mM solution of SA had 84 times more *PR1*-like transcripts than control plants at 24 hours after treatment. We then monitored the *PR1*-like transcript accumulation patterns during the early colonization stages of *Brachypodium* plants by *B*. *subtilis* B26. No difference in *PR1* transcripts accumulation could be detected for most of the post inoculation time points tested (S2 Fig). However, a modest and transient increase in *PR1* transcripts could be measured at 72 and 96 h dpi (S2 Fig). Taken together, this result suggests that *Bacillus subtilis* B26 is mostly perceived as a non-pathogenic bacterium during the systemic colonization of *Brachypodium distachyon*.

### Structural changes in colonized plant tissues

**Fig 3:**
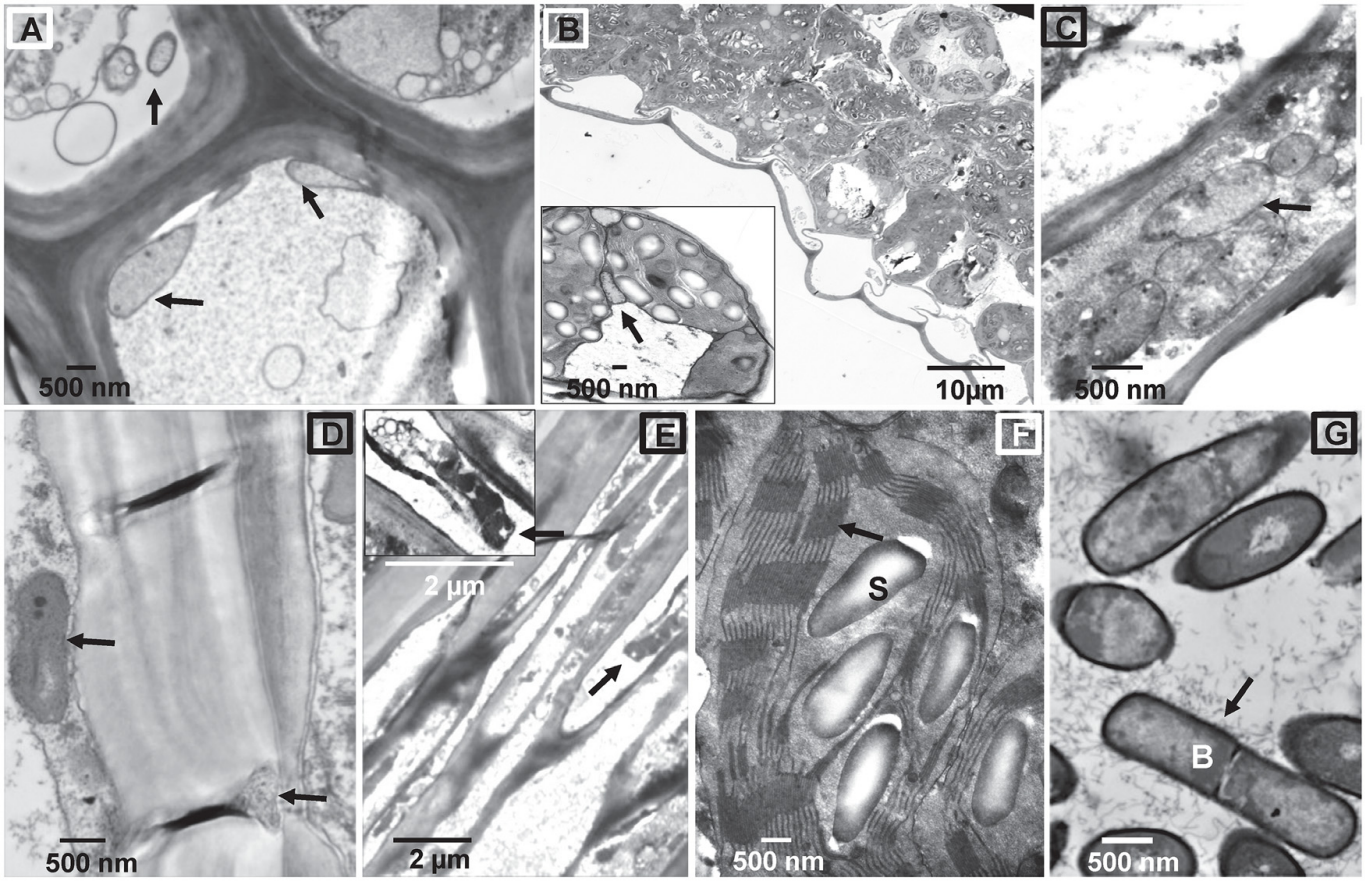
Transmission electron microscopy (TEM) micrographs of colonized *Brachypodium* tissues with *B*. *subtilis* B26. (A) Cross section of root xylem with numerous bacterial cells present inside the vessel elements (arrows). (B, C) Leaf mesophyll cells and bundle sheath (inset) with bacterial cells (arrows). (D) Vessel elements of xylem stem tissue showing B26 inside and outside the vessel elements. (E) Cross section of seed with B26 cells. (F) Cross section of chloroplast of a leaf bundle sheath cell from a colonized leaf. Notice the abundance of starch granules and the integrity of the thylakoids. (G) *B*. *subtilis* B26 cells grown in pure culture.

The interaction of *B*. *subtilis* B26 with *Brachypodium* was followed using transmission electron microscopy (TEM). We examined the internalization and distribution of *B*. *subtilis* B26 within roots, leaves, stems and seeds of colonized (14 and 28 dpi) *Brachypodium* plants grown under gnobiotic and greenhouse conditions (Fig 3). TEM analysis of tissue sections confirmed the presence of *B*. *subtilis* B26 cells inside xylem tissue of roots (Fig 3A), mesophyll cells and bundle sheath of leaves (Fig 3B and 3C) stems (Fig 3D) and also in seeds (Fig 3E). The morphology and size of *B*. *subtilis* cells inside plant tissues are identical to *B*. *subtilis* cells grown in pure culture (Fig 3G). Mesophyll cells close to leaf veins of colonized plants show substantial accumulation of unusually large starch granules in the chloroplast interspersed in the stroma and sometimes separating the thylakoids (Fig 3B and inset). However, the outer membranes of the plastids were still intact (Fig 3F, arrow). Mesophyll cells of non-colonized leaf blades had little or no starch granules (data not shown). Sections of control samples were devoid of bacterial cells (data not shown), suggesting no indigenous colonization.

### Colonized *Brachypodium* plants are more tolerant to water-deficit stress

**Fig 4:**
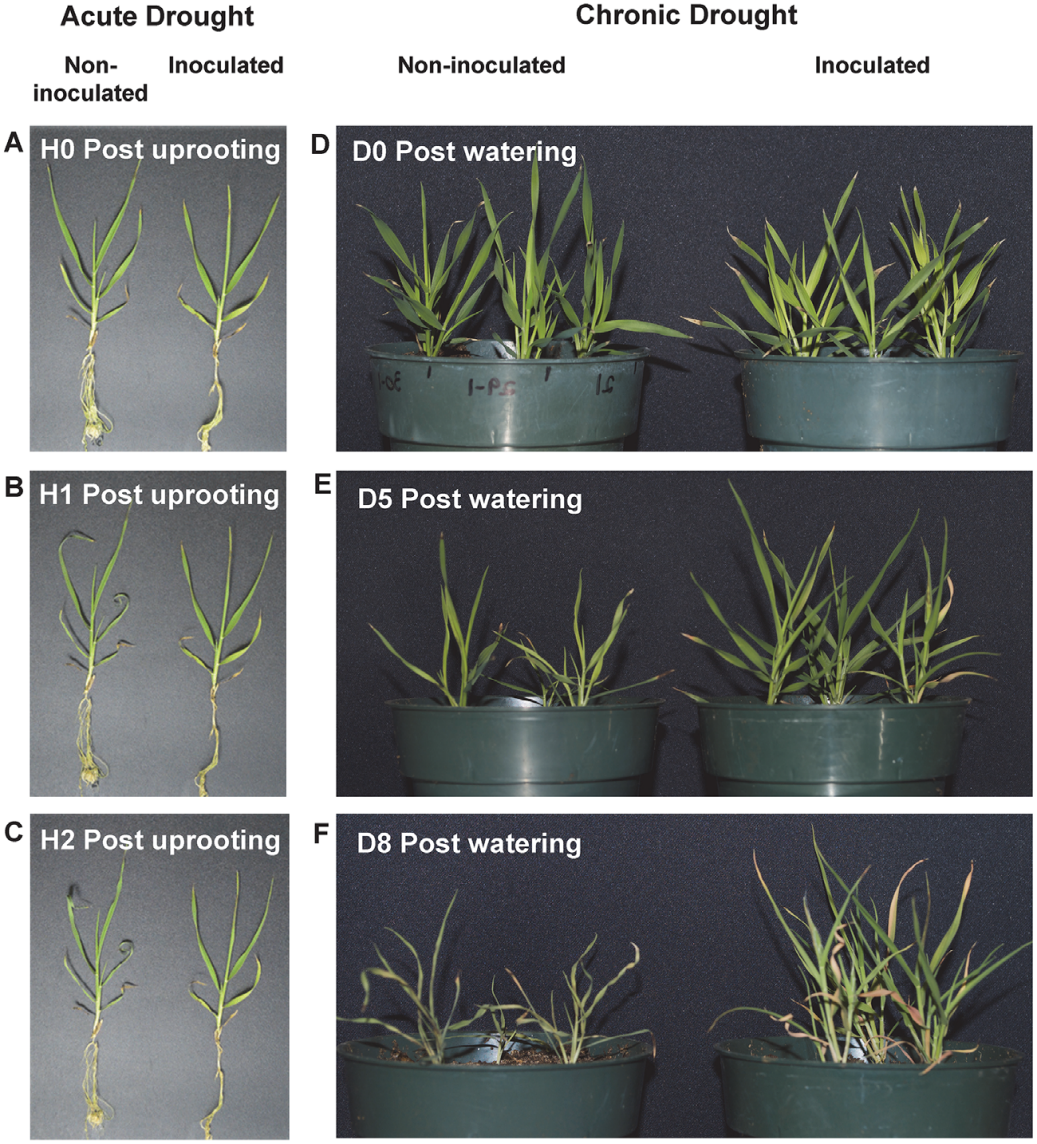
Effects of drought stress on non-inoculated and inoculated *Brachypodium* plants. Non-inoculated (left) and inoculated (right) *Brachypodium* plants (A) before or (B and C) after one and two hours of acute drought stress. Pictures of non-inoculated (left) and inoculated (right) *Brachypodium* plants were also taken at (D) 0 day, (E) 5 days and (F) 8 days after last watering.

An unexpected observation that inoculated *Brachypodium* plants uncared-for for several days were doing notably better than the non-inoculated ones prompts us to evaluate the contribution of *B*. *subtilis* B26 to the plant’s capacity to tolerate drought. Our initial assay consisted of an acute water-deficit stress applied by uprooting young non-inoculated and inoculated *Brachypodium* seedlings grown *in vitro* from the culture medium and leaving them on an open bench for 1h. The leaf tips of non-inoculated plants showed clear signs of wilting while inoculated plants looked mostly unaffected (Fig 4A–4C). We then performed a chronic drought treatment in a soilless potting mix with non-inoculated and inoculated plants at 28 dpi by withholding water for 5 and 8 days. Again, inoculated plants showed less signs of wilting and ultimately died later than non-inoculated plants (Fig 4D–4F).

### *B*. *subtilis* strain B26 modulates the expression of the plant’s drought responsive genes

**Fig 5:**
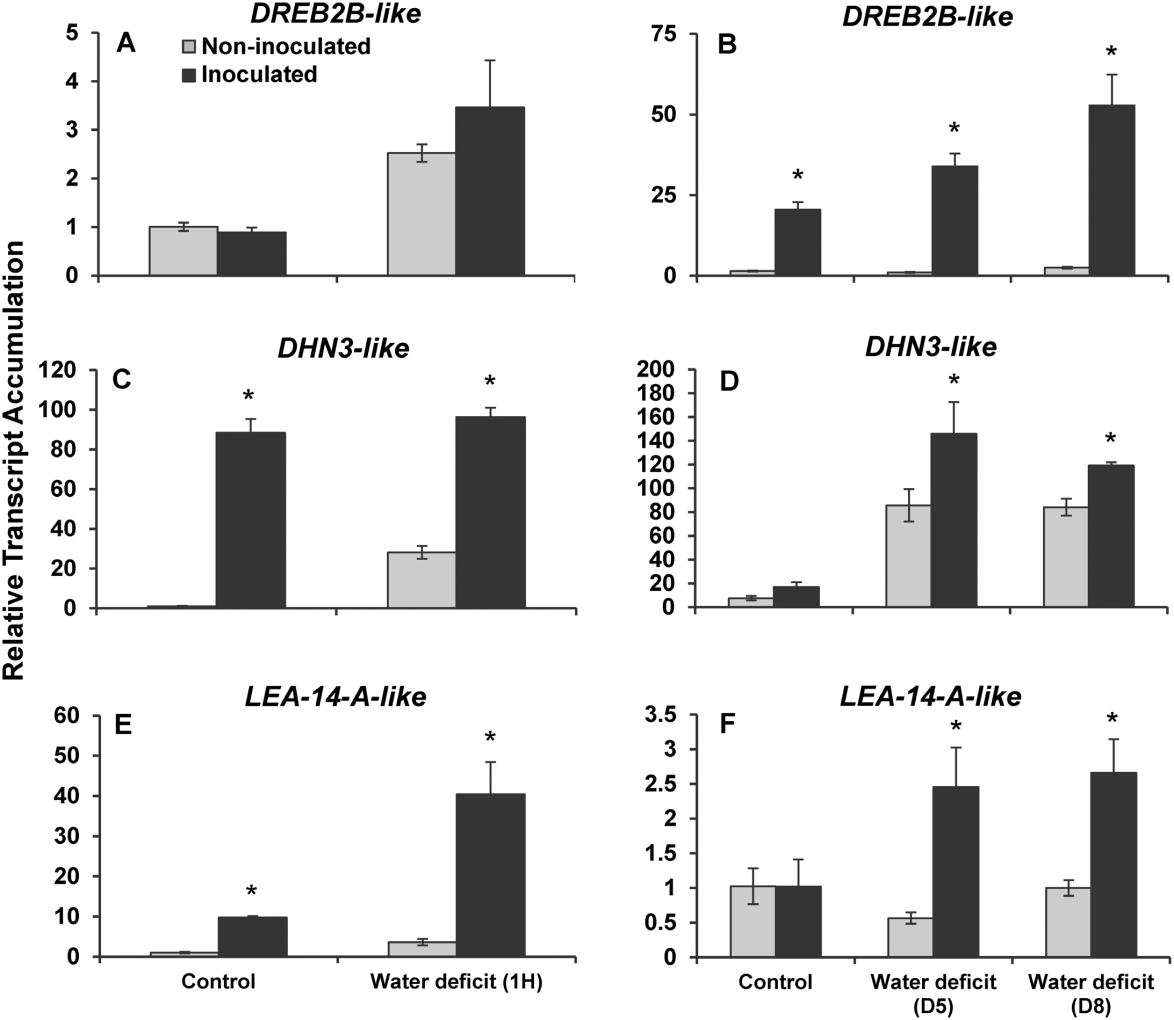
Relative transcript accumulation of drought-responsive genes. Relative mRNA abundance of (A, B) *Dehydration-Responsive Element-Binding protein 2B-like* (*DREB2B-like*); (C, D) *Dehydrin 3-like* (*DHN3-like*); and (E, F), *Desiccation-related protein LEA-14-A-like* in non-inoculated and inoculated plants before and one hour after uprooting (A, C, E) or before and after 5 and 8 days of chronic drought stress (B, D, F). * Represents a statistically significant difference.

To determine the role of *B*. *subtilis* B26 in the plant’s drought-response mechanism, we selected *Brachypodium* genes with high sequence similarities to genes previously characterized to play active roles in the drought-stress response of plants (S1 Table) and conducted quantitative real-time PCR assays to monitor their transcript accumulation profiles. Inoculated and non-inoculated *Brachypodium* plants grown *in vitro* under control conditions displayed similar accumulation profiles of the *DREB2B-like* transcript (Fig 5A). However, a one-hour acute drought treatment triggered increases in *DREB2B-like* transcripts accumulation of respectively 2.5 fold and 3 fold in non-inoculated and inoculated *Brachypodium* plants (Fig 5A). On the other hand, inoculated plants grown under normal conditions in soilless potting mix had 14-times more *DREB2B-like* transcript levels than non-inoculated plants grown under similar conditions (Fig 5B). In addition, chronic drought conditions, obtained by withholding water for 5 and 8 days, caused significant increases in the levels of *DREB2B-like* transcripts in inoculated plants while only a 1.7-fold increase was observed in non-inoculated plants (Fig 5B and S3 Fig).

The transcription factor DREB2B has been shown to act upstream of structural proteins such as dehydrins in *Arabidopsis* and other plants [25]. We thus sought to monitor changes in the expression profiles in response to acute and chronic drought stresses of two *Brachypodium* genes with high sequence similarities to the dehydrins *DHN3* and *LEA-14-A*. Compared to non-inoculated *Brachypodium* plants, a 70-fold accumulation in *DHN3-like* transcripts was observed in inoculated control plants grown *in vitro* (Fig 5C) while no significant difference was observed for plants grown in soilless potting mix (Fig 5D). The application of an acute drought treatment triggered a 20-fold accumulation of the *DHN3-like* transcript in non-inoculated plants but had no significant effect on the already high accumulation of this transcript in inoculated plants (Fig 5C). Conversely, chronic drought treatments of either five or eight days triggered a 85-fold accumulation of the DHN3-like transcript in inoculated plants and a 9-fold accumulation of the same messenger in non-inoculated plants (Fig 5D). A similar transcript accumulation pattern was also observed for the *LEA-14-A-like* gene (Fig 5E and 5F).

### *Bacillus subtilis* B26 stimulates carbohydrate and starch accumulation under drought stress conditions

**Fig 6:**
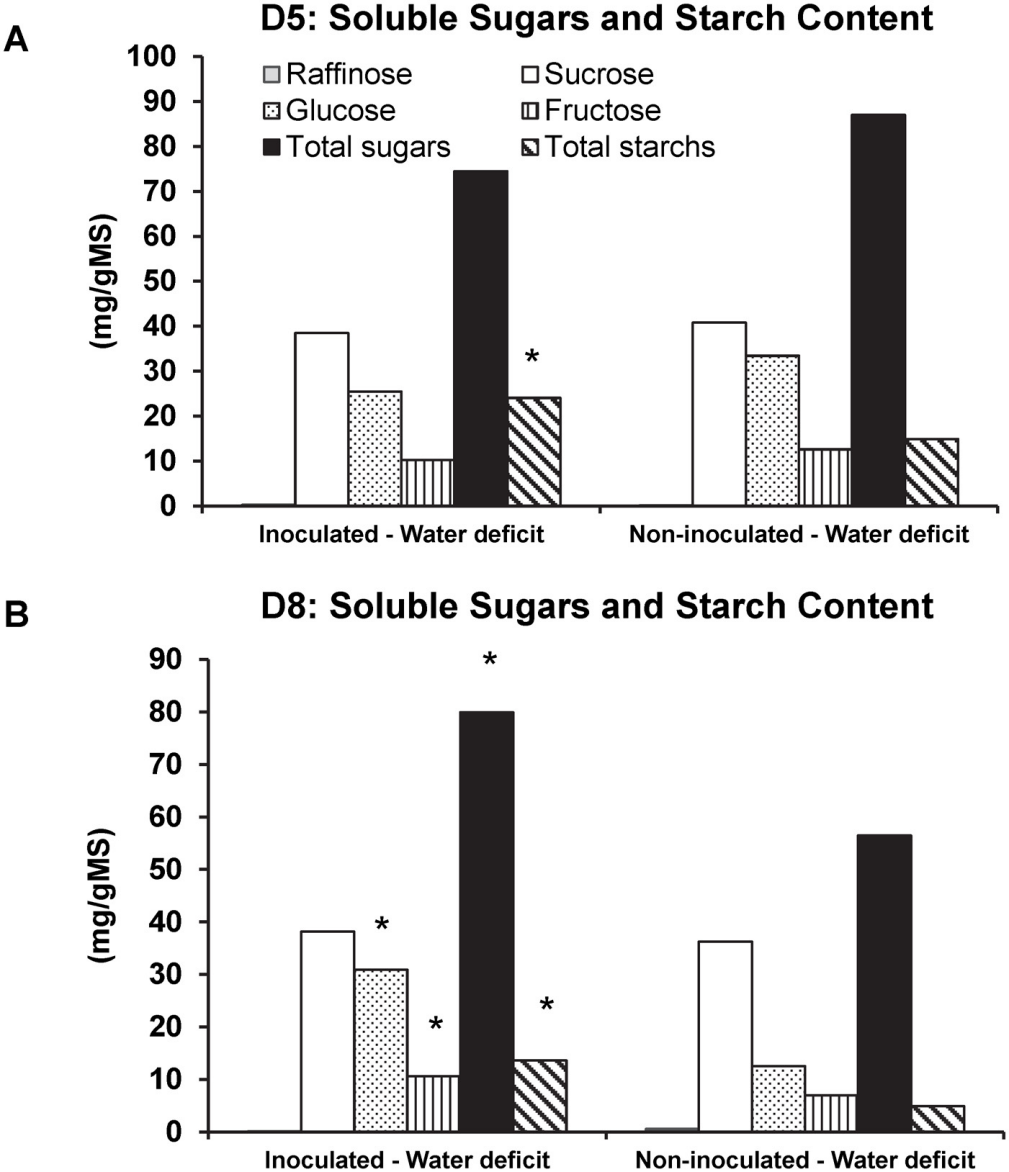
Soluble sugars and starch content of inoculated and non-inoculated plants under water deficit conditions. (A) 5 days and (B) 8 days post watering. * Represents a statistically significant difference.

Leaf tissues of inoculated and non-inoculated *Brachypodium* were analyzed for carbohydrate and starch content at the end of 5 and 8 days of chronic drought stress. Stressed inoculated plants had almost 2-fold and 3-fold increase of total starch at the end of 5 and 8 days of drought stress respectively, compared to stressed but not-inoculated plants (Fig 6). Drought stress did not have any influence on the concentration of individual and total sugars of inoculated and non-inoculated plants after 5 days of stress (Fig 6A). Inoculated plants exposed to stress for 8 days had 1.4-fold more of total soluble sugars, and also 2.9-fold and 1.4-fold increase in glucose and fructose concentrations, respectively (Fig 6B).

### *Bacillus subtilis* B26 triggers changes in DNA methylation in *Brachypodium*

**Fig 7:**
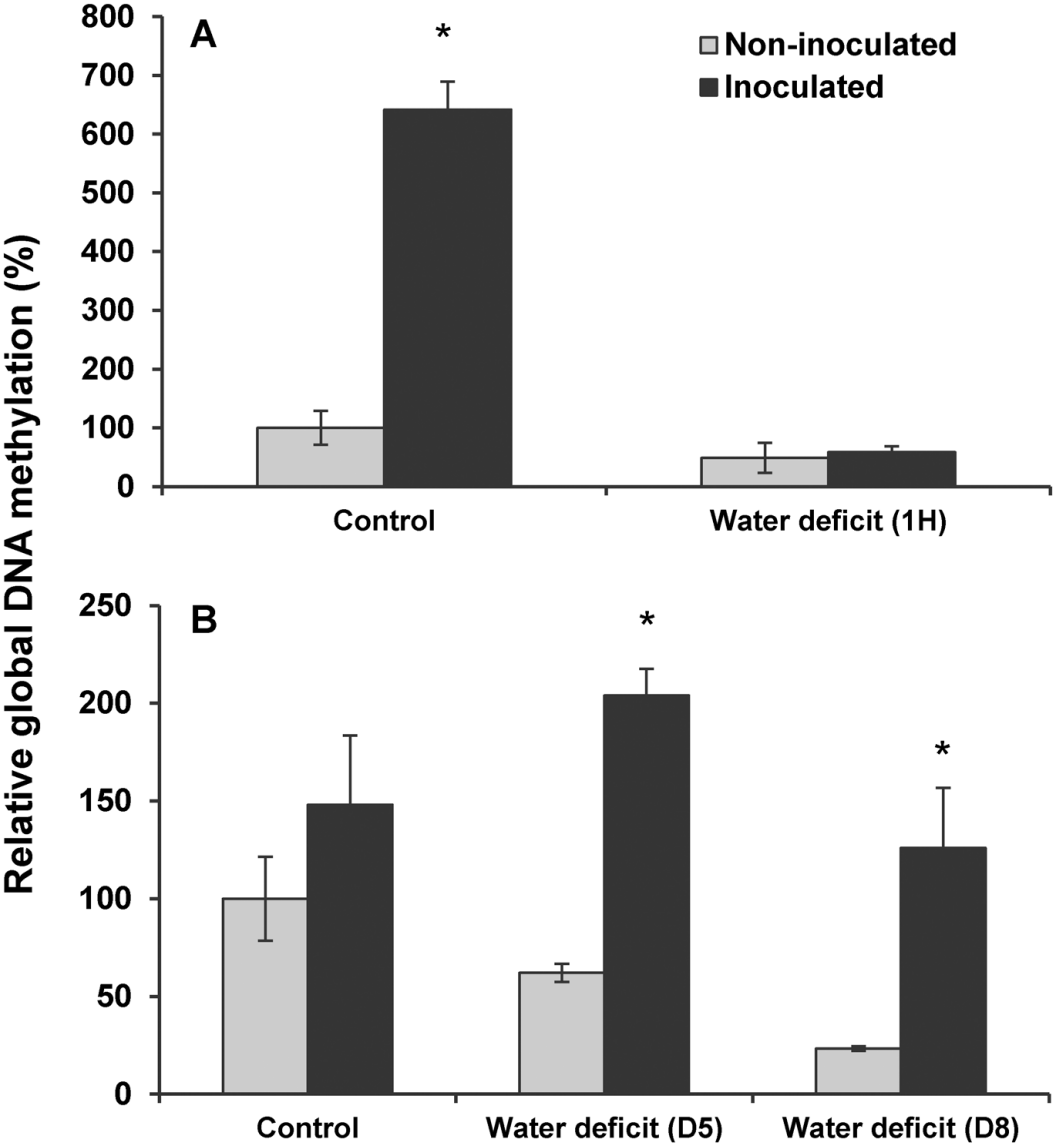
Variation of global DNA methylation in inoculated and non-inoculated *Brachypodium* plants under control and water deficit conditions. (A) Before and after one hour of acute drought stress. (B) Before and after 5 and 8 days of chronic drought stress. * Represents a statistically significant difference.

The changes in transcript accumulation observed in Fig 5 suggest that *B*. *subtilis* B26 triggers important chromatin changes in the host plant. We thus measured global DNA methylation in inoculated and non-inoculated *Brachypodium* plants under normal and drought conditions (Fig 7). *B*. *subtilis* B26 triggered 6-fold and 1.5-fold increases in global DNA methylation in plants grown under normal conditions either *in vitro* (Fig 7A) or in soilless potting mix (Fig 7B). On one hand, after one hour of acute drought treatment, the global DNA methylation levels observed in *in vitro* inoculated plants returned to those of non-inoculated plants while this treatment had no effect on the global DNA methylation levels of non-inoculated plants (Fig 7A). On the other hand, clear reductions in global DNA methylation were observed in non-inoculated plants after five and eight days of chronic drought treatment (Fig 7B). These reductions were not observed in inoculated plants exposed to similar drought stress conditions since an overall increase in global DNA methylation was observed after five days of chronic drought. These results suggest that *B*. *subtilis* B26 can affect the epigenetic regulation of *Brachypodium distachyon* before and during drought stress.

**Fig 8:**
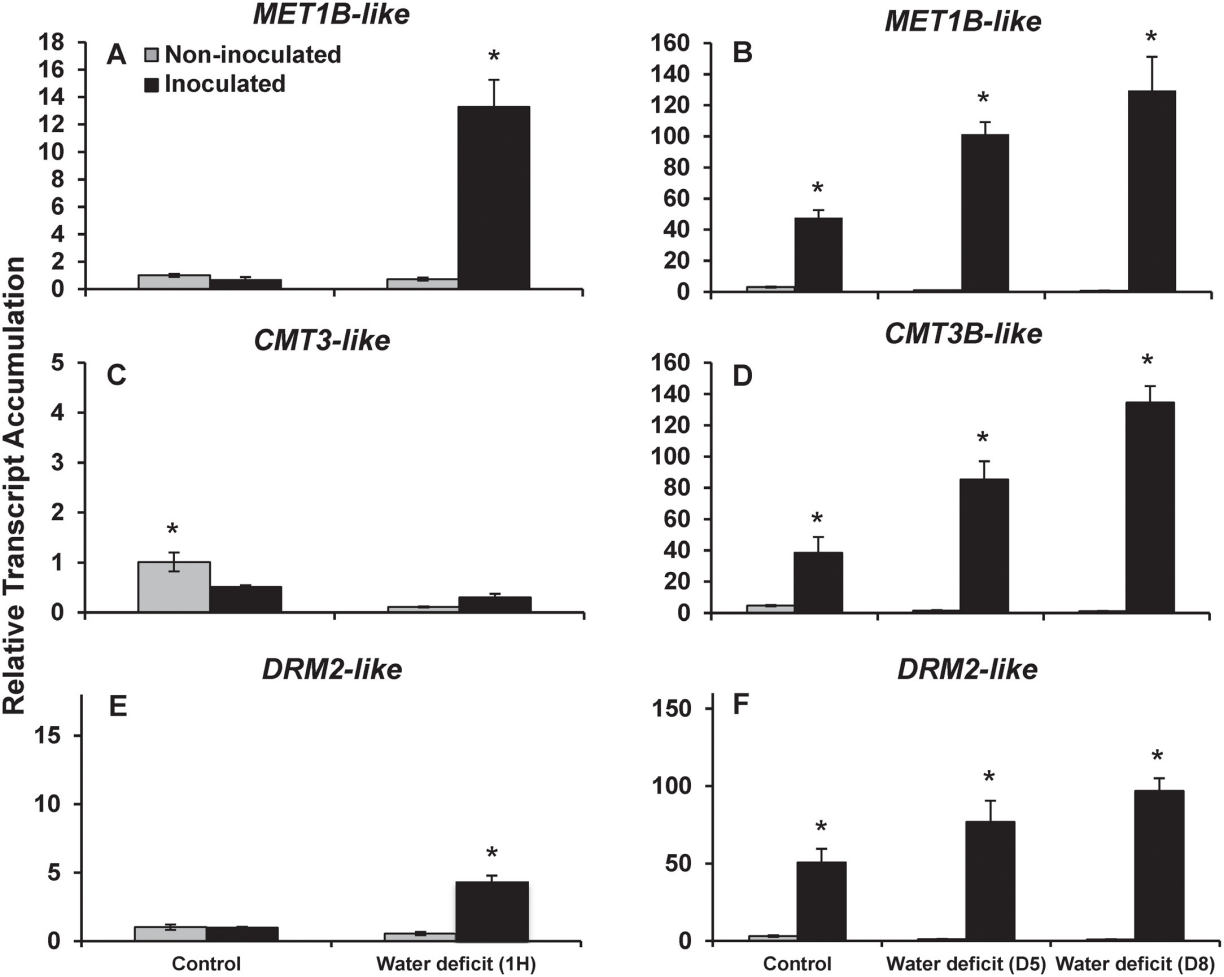
Relative transcript accumulation of DNA methyltransferases in inoculated and non-inoculated *Brachypodium* plants under control and water deficit conditions. (A, B) Relative mRNA abundance of *Methyltransferase 1* (*MET1*), (C, D) *Chromomethylase 3* (*CMT3*), and (E, F) *Domains-RearrangedMethyltransferase 2* (*DRM2*) before and one hour after uprooting non-inoculated and inoculated plants (A, C, E) or before and after 5 and 8 days following the last watering of non-inoculated and inoculated plants (B, D, F). * Represents a statistically significant difference.

The drastic changes in global DNA methylation observed upon colonization of *Brachypodium* suggest the involvement of several DNA methyltransferases in regulating that process. We thus monitored transcript accumulation changes in inoculated and non-inoculated plants in response to drought for three DNA methyltransferases: *MET1B-like*, *CMT3-like* and *DRM2-like*. As shown in Fig 8, drought treatments had very little impact on the transcript accumulation of the three DNA methyltransferases tested in non-inoculated plants either grown *in vitro* (Fig 8A, 8C and 8E) or in soilless potting mix (Fig 8B, 8D and 8F). Similarly, inoculated *Brachypodium* plants grown *in vitro* under control conditions did not show significant differences in accumulation of DNA methyltranferase transcripts (Fig 8A, 8C and 8E). On the opposite, inoculated *Brachypodium* plants subjected to one hour of acute drought stress showed increased *MET1B-like* and *DRM2-like* transcript accumulations (Fig 8A and 8E). In addition, inoculated plants grown in soilless potting mix under control conditions accumulated more of the three DNA methyltransferase transcripts than non-inoculated plants (Fig 8B, 8D and 8F). Moreover, chronic drought conditions for five and eight days further increased the accumulation of these transcripts in inoculated plants but not in non-inoculated plants (Fig 8B, 8D and 8F)

## Discussion

The present study was aimed to determine the potential for *Brachypodium distachyon* to act as a model to the PGB, *Bacillus subtilis*, and also to ascertain whether this interaction might serve as functional model to study at the molecular level how plant genes of cereals and perennial bioenergy grasses are modulated by the presence of bacterial endophytes and the genes expressed provide clues as to the effects of endophytes in grasses. The results clearly demonstrate the compatibility of an intimate interaction between *Brachypodium* and *Bacillus*, which is of greatest relevance as a PGB and inducer of abiotic stress tolerance [11,26–28].

*B*. *subtilis* strain B26 proved to be highly compatible to *Brachypodium* growth stages. In pot experiments and under a gnotobiotic environment with all environmental parameters controlled, a single event of soil drenching during the seedling stage promoted both root and shoot growth, increased plant height and number of leaf blades, and remarkably promoted seed yield compared to the untreated plants. Numerous studies have reported on PGBs, particularly on *Bacillus* spp. exerting a number of characteristics enabling to mobilize soil nutrients and synthesize phytohormones leading to plant growth promotion [8,12,29–33]. Indeed, growth stimulation of *Brachypodium* is probably related to the production of indole-3- acetic acid (IAA) and the cytokinin zeatin riboside by strain B26 [19]. Growth stimulation by bacterial endophytes has also been related to phosphorus mobilization [31]. Our strain B26 is known to solubilize phosphorus [19], however in this study, the experimental design did not allow to test for P solubilisation since all plants were fertilized with readily available concentrations of NPK. Nutrient content of above ground tissues were significantly lower in inoculated plants compared to control plants 56 days after treatment when plants reached the late milk stage (BBCH77) of seed development. Translocation of NPK from vegetative tissue to grain development in cereal grains has been reported [34,35], which may explain the overall reduction of NPK in vegetative tissues of inoculated plants and the copious numbers of seeds produced compared to control plants.

The ability of bacterial endophytes to colonize plants is a complex process requiring resistance to plant defence systems as well as the ability to initiate growth on plant surfaces, and develop internally inside the plant [32]. Indeed, *B*. *subtilis* B26 became intimately associated with *Brachypodium* since B26 could be isolated in reasonably high titres from rhizospheric soil, and surface sterilized roots, stems, leaf blades almost two months after initial treatment of *Brachypodium* young seedlings. Moreover, vertical transmission of B26 from one generation to the next via the seeds was confirmed by culture-dependent and independent methods of young seedlings derived from surface sterilized seeds and grown in gnotobiotic environment. Presence of bacterial endophytes in vegetative and reproductive plant tissues has also been described for other bacterial endophytes with plant growth promoting effect [13,32,36–38]. In the rhizosphere soil strain B26 exhibited stable population densities ranging from log 3.63 to 3.68 log CFU per gram of soil after the onset of inoculation and maintained them over *Brachypodim* growth stages. These densities are comparable to what had been reported for bacterial endophytes [39].

Although not frequently investigated, it is well known that endophytes may spread systemically inside plants and colonize stems, leaves [12]. DNA copies and cultivable population densities of strain B26 inside roots and shoots increased over time with tendencies for B26 to accumulate more in the above ground tissues than in roots. Comparable cultivable population densities were reported for *B*. *subtilis* strains in roots and leaves of wheat and mulberry [18,25,40].

Reports concerning the presence and role of bacterial endophytes in flowers, fruits and seeds are less numerous [14,32]. Interestingly, first generation seeds of *Brachypodium* harboured a small *B*. *subtilis B26* population density of 2.47 log. This is not surprising, as it is known that endophyte densities of the same species decrease during seed dormancy [41]. An increase in population density of B26 in roots and above ground tissues of young plants grown from these seeds, and attaining similar densities in *Brachypodium* of the same growth stage as those of the first generation is a strong indication that vertical transmission has occurred. Confirmation of the vertical transmission of B26 was obtained by culture-independent methods using strain-specific primers. The existence of vertical transmission of strain B26 is very interesting as it enables a plant with an established endophytic community to pass bacteria with beneficial characteristics to their offsprings, and ensures the presence at early stages of seedling growth [42].

True endophytic bacteria are recognized by their capacity to re-infect disinfected seedlings and by establishing visualized evidence of their localization inside plant tissues [43]. In this study, we have fulfilled both criteria. Systemic spread of the endophyte within the roots, leaves and seeds was successfully confirmed by culture-dependent and independent methods and the visualization and spread of B26 inside plant tissues confirms that they have moved from the roots and travelled upward to the stem and leaves. The exact localization of *B*. *subtilis* in aerial plant tissues was investigated by transmission electron microscopy (TEM). Cells of *B*. *subtilis* strain B26 were visualized and their migration and inter- and intracellular colonization of vegetative and reproductive tissues of *Brachypodium* by strain B26 was confirmed. The ability to colonize the intercellular spaces near the vascular bundles shows that strain B26 can traverse the endodermis in roots. It is likely that strain B26 cells were able to pass through the endodermis and can secrete cell wall degrading enzymes allowing them to continue colonization inside the root [44] or alternatively may have passed passively during secondary root formation when the endodermis is often disrupted [45]. Following colonization of the root interior, strain B26 spread to the stems, leaf blades and seeds, is most probably via the perforated plates of the xylem vessels or by colonizing intercellular spaces form roots to aerial parts as commonly reported for other endophytes [46,47]. However, presence of B26 inside mesophyll cells surrounding leaf bundle sheath is a strong indication of intracellular colonization. How B26 cells were able to pass over several structural and cellular barriers is not known and remains to be unravelled. To exit xylem cells, strain B26 may cause rupture of the cell wall by chemical dissolution of primary and secondary walls. Strain B26 is a strong producer of cellulases [19] reinforcing the notion that the bacterium secretes cell wall degrading enzymes that will soften cell wall, thus facilitating the progression of the endophyte towards adjacent cells.

Drought is one of the most important abiotic stress limiting crop growth and productivity [48]. Studies on systemic tolerance to drought reported that inoculation with plant growth promoting rhizobacteria enhanced drought tolerance via the increased transcription of drought-response genes [49], affecting the phytohormonal balance [50] and sugar accumulation [51]. Here, we hypothesized that the establishment of strain B26 in the rhizosphere and roots of *Brachypodium* represents the first line of defense against drought stress. The second defense barrier against drought stress might constitute the endophytic colonization of plant tissues that enhance the plant’s response at the gene and biochemical levels.

The establishment of these lines of defense correlates well with an increase in expression of several *Brachypodium* genes associated with drought stress and changes at the epigenetic level as well as the accumulation of total soluble sugars and structural starch observed in inoculated *Brachypodium* plants. At the transcriptional level, B26 stimulated the induction of drought-responsive genes (*DHN3 and LEA-14A*) and also the transcription factor modulating dehydration responsive element binding gene (*DREB2B)* under acute and chronic water stress. Depending on the gene, accumulation of transcripts was more than 14 fold and reached in some cases as high as 85 in inoculated plants. We believe that systemic colonization of *Brachypodium* by strain B26 reduces the drought stress phenotype, thus aggravating the need to express drought-signalling response. Supporting evidence on the enhancement of transcripts of *DREB2*, dehydrins and other related drought-responsive genes in rhizobacteria-associated crops are provided by studies on sugarcane, mung beans and *Arabidopsis* [49,52–54].

Changes in DNA methylation in the presence of the plant growth promoting bacterium *Burkholderia phytofirmans* were recently reported in potato [55]. In this study, we demonstrated that the colonization of the *B*. *subtilis* B26 caused an increased in the abundance of methyltransferases involved in the maintenance and regulation of DNA methylation and a hypermethylation of *Brachypodium*’s genome. We further showed that during chronic drought stress, the inoculated plant’s global DNA methylation levels remained high when compared to those of non-inoculated plants suggesting that *B*. *subtilis* B26 could potentially act at the epigenetic level to increase drought stress tolerance in *Brachypodium*. This agrees well with the fact that drought conditions have been shown to naturally induce DNA methylation changes in the plant [56] that in turn increase the plant resistance toward the stress by allowing the expression of protective genes involved in the drought response.

Osmoregulation in plants via accumulation of soluble sugars like glucose, sucrose and fructose is a known mechanism for maintaining homeostasis in plants under drought stress conditions [57] and their metabolism play a significant role in drought and cold stress tolerance [57,58]. Imposition of drought stress to *Brachypodium* significantly increased total soluble sugar and starch in above ground tissues of *Brachypodium*-inoculated plants, which in turn could compensate for the drought effects and improve plant developments through among others, the enhanced production of soluble sugars resulting in a better absorption of water and nutrients form the soil. Similarly, increased biosynthesis rates of soluble sugars in corn inoculated with a plant growth promoting *Pseudomonas* exposed to drought stress was also reported [51]. Incidentally, drought stressed and inoculated plants accumulated more starch than control stressed plants. The greater amount of starch in these plants might be related to increased availability of photosynthates for storage in leaves during drought.

The latter observation ties well with copious accumulation of large starch granules in the stroma of chloroplasts of leaf bundle sheath cells of inoculated plants relative to control plants. The starch packing had no visible effects on the grana. To the best of our knowledge, this extensive loading of leaf chloroplasts with starch in response to bacterial endophytic colonization has not been reported. In addition to increased availability of starch as reserve to plants under stress, this modification could result in the enhancement of nutrient flow to bacterial cells, however more work is required to understand the effects of this starch accumulation.

## Conclusions

Understanding the mechanisms behind PGB-plant interactions is important to improve strategies for the use of PGB in agriculture. Here, we provide evidence that endophytic colonization of *Brachypodium* by *B*. *subtilis* strain B26 affect the whole cycle of a plant, accelerating its growth and shortening its vegetative period. Also we demonstrate that *B*. *subtilis* strain B26 is able to colonize and multiply in the upper portion of the plant and is vertically transmitted from one generation to the next via seeds. Also, we described that strain B26 confers resistance against drought stress in *Brachypodium* and this is linked to the upregulation of expression of several drought-responsive genes and the modulation of the DNA methylation process. Overall these findings contribute to a better understanding of plants and beneficial bacteria interactions in grasses and cereal crops, and provide novel information on the long-term effect of PGB on plant development.

## Methods

### Maintenance and preparation of *Bacillus subtilis* B26 inoculum

The *Bacillus subtilis* strain B26, previously isolated from leaves of switchgrass cultivar Cave-in-Rock grown under field conditions, and fully characterized [19], was maintained on Luria Broth (LB) (1.0% Tryptone, 0.5% Yeast Extract, 1.0% NaCl) (Difco, Franklin Lakes, NJ, USA) with glycerol (25% final volume) and stored at -80C. *B*. *subtilis* B26 was revived on LBA (1.5% Agar) (Difco) plates. Inoculum was prepared by placing a single colony of *B*. *subtilis* B26 in 250 ml of LB and incubated for 18 h at 37°C until an OD600 of 0.7 was reached on a shaker at 250 rpm to the mid-log phase, pelleted by centrifugation, washed and suspended in sterile distilled water [19].

### *Brachypodium* line, growth conditions and *B*. *subtilis* inoculation

#### Growth Chamber Experiments

*Brachypodium distachyon* plants from the inbred line Bd21 [22] were used throughout. Bd21 seeds were surface sterilized by sequentially immerging them in solutions of 70% ethanol for 30 seconds and 1.3% solution of sodium hypochlorite for 4 minutes before rinsing them three times in sterile water [59]. Cone-tainer (Stuewe and Sons, Tanent, OR, USA) of 164 ml capacity were used to grow the plants. Prior to use, Cone-tainers were surface sterilized for 12 h in 0.1% NaOCl and rinsed with distilled water. Each Cone-tainer was filled with 1:1:1 part of sand (Quali-Grow, L’orignal, ON, Canada)/perlite (Perlite Canada, Lachine, QC, Canada)/Agro Mix PV20 (Fafard, Saint-Bonaventure, QC, Canada) previously autoclaved for 3 h at 121°C on three constitutive days. Three Bd21 sterile seeds were planted in each Cone-tainer and stratified at 4°C for 7 days after which they were placed in a climatically controlled chamber (Conviron, Winnipeg, MB, Canada) under a 16–h photoperiod with a light intensity of 150 μmoles/m^2^/s and a day/night temperature regime of 25/23°C. Plants were watered three times per week with sterile distilled water and fertilized every 14 days with 40 ml of a solution of 2 g/liter of N-P-K fertilizer 20-20-20 (Plantprod, Laval, QC, Canada) per Cone-tainer. Plants were thinned to two per Cone-tainer after 14 days of growth, and at the same time each Cone-tainer received 5 ml of *B*. *subtilis* B26 inoculum (10^6^ CFU/ml) or 5 ml of water (control). Inoculated and non-inoculated (control) Con-tainers were placed in growth chambers with identical growth parameters as previously described. Plants were harvested after 14, 28, 42 56 days post inoculation (dpi). Seeds collected from inoculated plants 56 days post inoculation were planted following the same growth conditions except that that they were not reinoculated with B26. Second generation plants were harvested after 28 days of growth.

#### *In-vitro* Culture Experiments

plant were grown in disposable culture tube 25 X 150 mm (VWR, Radnor, PA, USA) in 1X Murashige and Skoog medium with 0.3% sucrose supplemented with GAMBORG’ vitamins (Sigma-Aldrich Corp., St. Louis, MO, USA). Stratification, seed sterilization, growth conditions and inoculation were performed in a similar manner as those grown in growth chambers. Plants were inoculated with 5 ml of *B*. *subtilis* B26 after 10 days of growth. Control plants received 5 ml of sterile distilled water

### Monitoring of growth parameters of Bd21 line

Fourteen-day-old test and control Bd21 plant groups grown in controlled growth chambers were harvested at defined phenological growth stages (Table 1) using the BBCH numerical scale [24]. Harvesting was done at growth stage BBCH 13 prior to inoculation with *B*. *subtilis* B26 (i.e., 0 dpi) and at the following dpi with their corresponding growth stage 14 dpi (BBCH45), 28 dpi (BBCH55), 42 dpi (BBCH77), 56 dpi (BBCH97). At each harvesting time point, a minimum of fourteen Bd21 plants from seven inoculated and non-inoculated Cone-tainers were monitored for root and shoot lengths, shoot and root dry weights, and number of leaves and tillers. Spikelet formation was recorded on a weekly basis while the number of seeds heads and viable seeds were recorded at the end of each experiment. Above ground nutrient content of N, P, K and Mg in vegetative above ground tissues was analyzed by Kjeldahl procedure using sulphuric acid and hydrogen peroxide digestion [60]. Values were estimated in mg per gram of dry weight of tissue. All experiments were replicated two times using different growth chambers in order to control the effects of microenvironment variation.

### Growth conditions and drought stress

To investigate whether *B*. *subtilis* B26 confer drought tolerance to Bd21, two types of drought stress were applied: chronic and acute water deficit stresses. Studies on the effect of chronic water deficit stress were carried out on *Brachypodium* seedlings stratified and germinated as previously described but planted in 10 x 10 cm pots (ITML, Brantford, ON, Canada) filled with sterilized Agro Mix G6 (Fafard, QC, Canada) with three plants per pot. Plants were grown under the same growth chamber conditions and were inoculated or not with *B*. *subtilis* B26 as previously described. Chronic water deficit stress was conducted on test and control plants at dpi 28 by withholding water from the inoculated plants while control plants were watered with 150 ml of sterile water 3 times per week. Plants were harvested on day 0, 5 and 8 of withholding water and leaf tissue was immediately frozen in liquid nitrogen and prepared for transcript accumulation analysis for drought responsive genes and starch and sugar content analysis. A total of 3 replicates per treatment were sampled at each time point. A replicate consisted of 3 plants. The experiment was repeated twice.

Acute water deficit stress was applied on young Bd21 seedlings grown in vitro cultures at 3 dpi, by uprooting the plants from the medium and left on an open bench for 1 hour before being flash frozen in liquid nitrogen. The entire plants were sampled, flash frozen in liquid nitrogen and subjected to transcript accumulation analysis. A total of 4 replicates per treatment were sampled and the experiments were repeated three times.

### Distribution and colonization of *Brachypodium* by *B*. *subtilis* B26 using culture-dependent and culture-independent methods

To ensure that *B*. *subtilis* B26 successfully and systemically colonized different plant tissues of the accession Bd21 and its intracellular spread is sustained at various *Brachypodium* growth stages (i.e., early and late vegetative, and reproductive stage), bacteria cell numbers and DNA copy number were determined in tissue samples and rhizosphere soil of inoculated and control *Brachypodium* plants. Root and leave tissues of test and control plants (first generation) of different growth stages were sampled at 14, 28 and 42 dpi, and entire young *Brachypodium* plants from second generation were sampled at 28 days of growth. All plants were surface sterilized as previously described [19]. 200 mg of tissue were pulverized to powder using a sterile mortar and pestle, serially diluted in sterile distilled water and plated on LBA. Bacterial enumeration of rhizosphere soil (1 gram) from inoculated and control *Brachypodium* plants was performed by serial dilution in sterile distilled water, shaken for 30 min and plated on LBA [61]. Plates were incubated at 37°C for 48 h. Colony forming units (CFUs) were determined and calculated to CFU per gram of fresh weight of tissue or soil. There were three biological replicates for each treatment and each replicate contained root, aerial systems or rhizosphere soil of 3 plants.

The presence of *B*. *subtilis* B26 cells inside inoculated plants was also confirmed by quantitative real-time PCR (qPCR) assays. Surface sterilized plant tissues were reduced to powder in liquid nitrogen, and genomic DNA was extracted from 200 mg of powdered tissue using the CTAB method [62] and resuspended in 100 μL of autoclaved distilled water. Genomic DNA from *B*. *subtilis* B26 colonies was extracted by direct colony PCR [63]. Briefly, single colonies were mixed with sterile distilled water, incubated at 95°C followed by centrifugation and the supernatant was used as template DNA in conventional PCR assays.

### Transmission electron microscopy of endophytic colonization by *Bacillus subtilis* B26

Fresh plant organs (roots, stems, leaves), removed from inoculated and their corresponding plants grown *in vitro* and in potting mix in growth chambers, were collected 5 days and 14 dpi days after inoculation, respectively. In parallel, seeds collected from the first generation plants were also collected. Sample were processed following the protocol by Wilson and Bacic [64] but with some modifications: fixation was carried out with 2.5% glutaraldehyde in 0.1M sodium cacodylate buffer for 7 days at 4°C, sample were washed 3 times with 0.1M sodium cacodylate washing buffer and finally an extra staining with tannic acid 1% staining was performed after the osmium tetroxide staining. After polymerization, capsules were trimmed and cut in section of 90–100 nm thick with an UltraCut E ultramicrotome (Reichert-Jung, Depew, NY, USA) and placed onto a 200 mesh copper grid. Samples were further stained with uranyl acetate for 8 min, followed by Reynold's lead for 5 min. Samples were observed using a FEI Tecnai 12 120 kV transmission electron microscope (TEM) equipped with an AMT XR80C 8 megapixel CCD camera (Hillsboro, OR, USA). All reagents were purchased from Electron Microscopy Sciences, Hatfield, PA, USA, except for the osmium tetroxide and Epon that were supplied from Mecalab, Montreal, QC, Canada.

### PCR amplification and quantification of *B*. *subtilis* B26 DNA copy number in inoculated plant tissues and seeds

The presence of *B*. *subtilis* strain B26 within vegetative and reproductive tissues of first and second generation *Brachypodium* plants was confirmed by PCR using strain-specific primers (S1 Table). PCR reactions along with no template controls were run under previously described conditions [19] using T100 Biorad thermal cycler (BioRad, Hercules, CA, USA). PCR products were separated on 1% agarose gels and visualized using Gel Logic 200 Imaging system from (Kodak, Rochester, NY, USA) under UV light.

Quantification of *B*. *subtilis* B26 DNA copy number as a measure of colonization of vegetative and reproductive organs of *Brachypodium* was monitored at different growth stages and also in second generation plants grown from inoculated seeds using qPCR. *B*. *subtilis* amplicons were purified with a QIAquick PCR-purification kit and cloned into pDrive (Qiagen, Venlo, Netherlands). Plasmid DNA was purified and sent for sequencing at Genome Quebec. Sequencing results were compared to the Genbank accession Ref#JN689339. The copy number of plasmid was calculated based on the concentration of purified plasmid DNA and the molecular mass of the plasmid (vector plus amplicon). A standard curve for *B*. *subtilis* B26 was constructed based on the following copy numbers: 10^9^, 10^8^, 10^7^, 10^6^, 10^5^, 10^4^, 10^3^ and 10^2^ which are the range of *B*. *subtilis* B26 copy numbers in the different tissues of the plant. The amplification mixture reaction contained: 400 ng of template DNA, 12.5μL of 2x SYBRII master mix (Agilent Technologies, Morrisville, NC, USA), 2.5 μmol L^-1^ of each primer and 2 μmol L^-1^ of ROX (Agilent Technologies) in a total volume of 25 μl. To overcome the effects of inhibitors present in the root DNA, 2.5 mg of BSA (Sigma-Aldrich) and 3% of DMSO (Fisher, Ottawa, ON, Canada) were added to each reaction. Amplification was performed in a Stratagene Mx3000P real-time thermal cycler (Agilent Technologies) under the following conditions: one cycle of initial denaturation at 95°C for 10 min, followed by 40 cycles of denaturation at 95°C for 30 s, annealing at 50°C for 45 s and extension at 72°C for 45 s. Standard curves and no template controls were run with each plate. All samples were performed in triplicate technical runs. Amplification results were expressed as the threshold cycle (Ct) value and converted to copy numbers by plotting the Ct values against the standard curve. The coefficient of variation was calculated for each sample to ensure repeatability of amplification. Samples with a coefficient of variation above 1 had their outliers removed.

### RNA extraction, cDNA synthesis

Aerial parts of four inoculated and non-inoculated plants were pooled and reduced to fine powder in liquid nitrogen. Total RNA was extracted from 100 mg of powder using the Total RNA Mini Kit, plant (Geneaid, Shanghai, China) following the manufacturer’s protocol. All RNAs were treated with DNase I (Qiagen) to remove genomic DNA (Qiagen) cDNA was synthesised using the iScript cDNA Synthesis Kit (BioRad). The resulting cDNA samples were diluted to a final concentration of 2.5ng/μL for qPCR, and stored at -20°C. Parallel reactions were run for each RNA sample in the absence of reverse transcriptase (no RT control) to assess any genomic DNA contamination.

### Gene identification and primer design

Using *Arabidopsis thaliana* protein sequences as query, identified *Brachypodium* distachyon’s orthologs of the following drought-responsive encoding genes; *DREB2B*, *LEA-14*, the defence encoding genes *PR1*, and the DNA methyltransferase encoding genes *MET1*, *CMT3*, and *DRM2* were used. The drought responsive gene, DHN3-like was identified using a DHN3 protein sequence from *Hordeum vulgare* (S1 Table). Primer sets were designed using Primer BLAST for specificity and synthesized by Integrated DNA Technologies, Inc. (Coralville, IA, USA). The primer pairs for 18S Ribosomal RNA and SamDC have been used previously [65,66].

### RT-QPCR data analysis and relative quantification of stress-responsive genes and PR1

Quantitative real-time PCR was performed using a CFX Connect Real Time system (BioRad), using Sso-advanced SYBR green Supermix (BioRad). Amplification was performed in an 11 μl reaction containing 1x SYBR Green master mix, 200 nM of each primer, 10 ng of cDNA template. The PCR thermal-cycling parameters were 95°C for 30 seconds followed by 40 cycles of 95°C for 5 seconds and 57.5°C for 20 sec (S1 Table). Three technical replicates were used and the experiment was repeated three times with different biological replicates. Controls without template were included for all primer pairs. For each primer pair, two reference genes (*18S* and *SamDC*) were used for normalisation. The RT-qPCR data was analysed following the Livak method [67].

### Global DNA methylation assay

The global DNA methylation assay was performed using the Imprint Methylated DNA Quantification Kit (Sigma-Aldrich) according to the manufacturer's recommendations with 200 ng/μL of DNA per well. Each sample was measured in technical quadruplicate using a 680 Microplate reader (BioRad). Genomic DNA was extracted following the methods mention previously.

### Starch and water-soluble sugar analysis

One hundred (100) mg of freeze-dried ground leaf tissues of inoculated or not plants subjected to drought or not were pooled and reduced to fine powder in liquid nitrogen. Soluble sugars were extracted with methanol/chloroform/water solutions and analyzed as described in Bertrand and coworkers, [68] using a Waters High Performance Liquid Chromatography (HPLC) analytical system controlled by the Empower II software (Waters, Milford, MA, USA). Peak identity and quantity of raffinose, sucrose, glucose and fructose were determined by comparison to standards. Total starch was extracted from the non-soluble residue left after the methanol/chloroform/water extraction and quantified as a glucose equivalent following enzymatic digestion with amyloglucosidase (Sigma-Aldrich) and colorimetric detection with ρ-hydrobenzoic acid hydrazide method of Blakeney and Mutton [69].

### Statistical analyses

All experimental data were subjected to statistical analyses by performing one-way ANOVA using the JMP 10.0 software (SAS Institute, Cary, NC, USA). The significance of the effect of the treatments was determined via Tukey HSD with a magnitude of the F-value (*P* = 0.05). In the case of repeated experiment trials results were tested using Levene’s test for equality of variance (*P* = 0.05) and pooled if permitted.

## Supporting Information

S1 Fig The effect of *B*. *subtilis* B26 on seed production of *Brachypodium distachyon* Bd21. (A) Seed head and (B) spikelet number. * Represents a statistically significant difference.
(TIFF)

S2 Fig Relative transcript accumulation of *PR1-like* gene in *Brachypodium distachyon* Bd21 plants. (A) Inoculated and non-inoculated plants from 0 to 168 hours post-inoculation with *B*. *subtilis* strain B26. (B) Accumulation of *PR1-like* in plants treated or not with Salicylic Acid (B).
(TIFF)

S3 Fig Relative transcript accumulation of *DREB2B-like* in non-inoculated *Brachypodium* plants under control and chronic drought conditions. Relative mRNA abundance of *Dehydration-Responsive Element-Binding protein 2B-like* (*DREB2B-like*) in non-inoculated plants before after 5 and 8 days of chronic drought stress. * Represents a statistically significant difference.
(TIFF)

S1 Table List of primers used in this study. (DOCX)

S2 Table Nutrient analysis of above ground of control (C) and inoculated *Brachypodium* with *B*. *subtilis* 26 (B+). (DOCX)
