# Supplementary figures and images for: Accelerated Growth Rate and Increased Drought Stress Resilience of the Model Grass Brachypodium distachyon Colonized by Bacillus subtilis B26
Source: PLoS One. 2015 Jun 23;10(6):e0130456. doi: 10.1371/journal.pone.0130456 (PMC4477885; doi:10.1371/journal.pone.0130456)

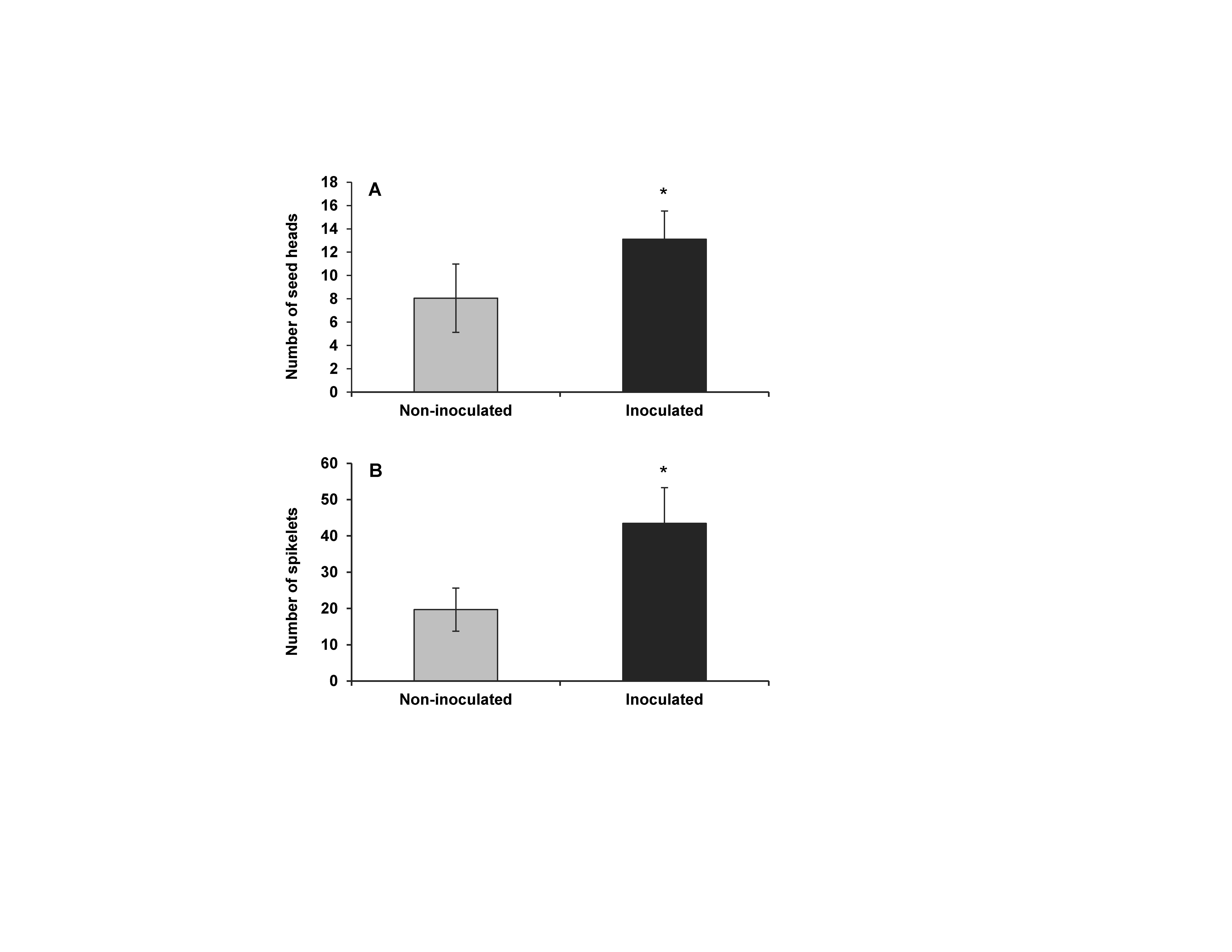

Supplement: S1 Fig — (A) Seed head and (B) spikelet number. * Represents a statistically significant difference. (TIFF) [file pone.0130456.s001.tiff]

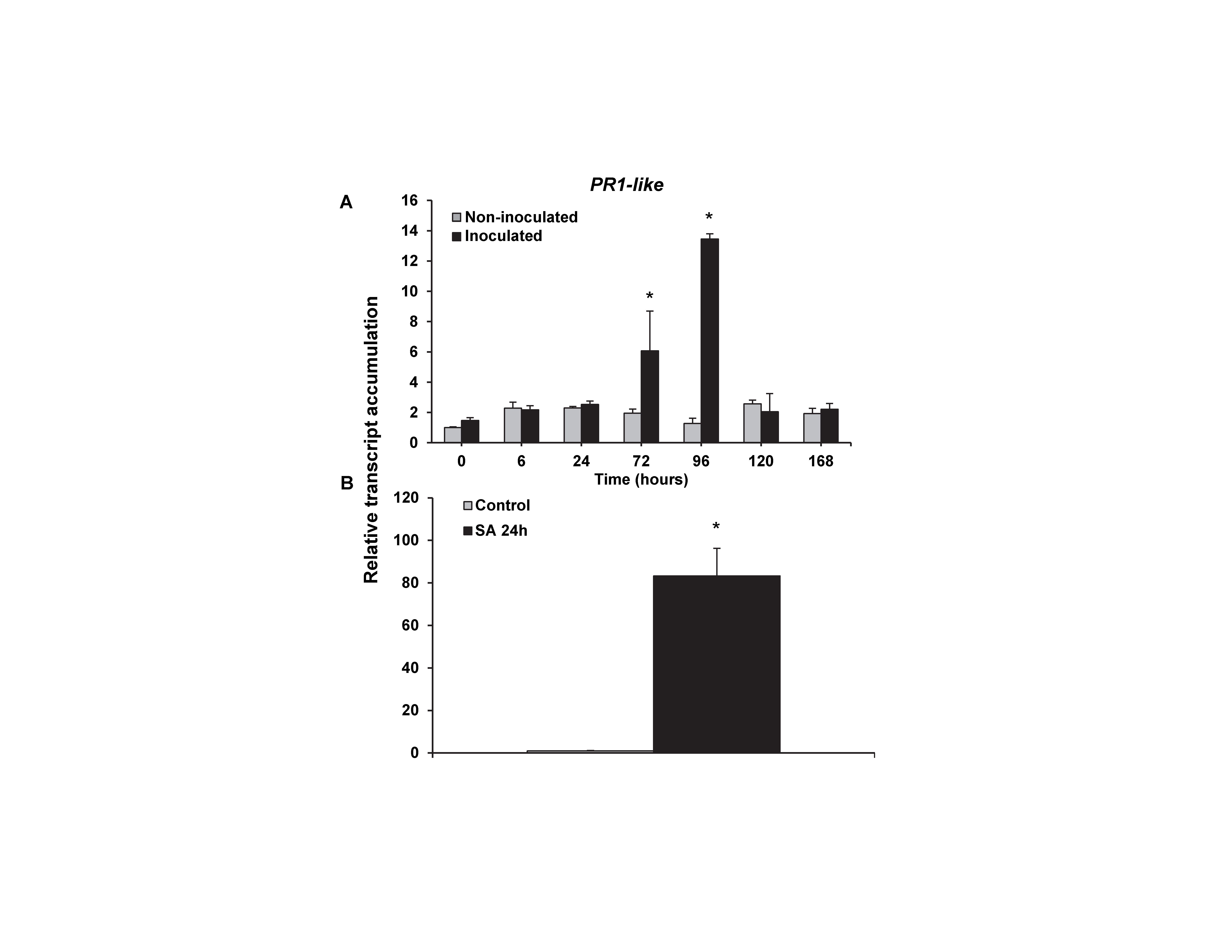

Supplement: S2 Fig — (A) Inoculated and non-inoculated plants from 0 to 168 hours post-inoculation with B. subtilis strain B26. (B) Accumulation of PR1-like in plants treated or not with Salicylic Acid (B). (TIFF) [file pone.0130456.s002.tiff]

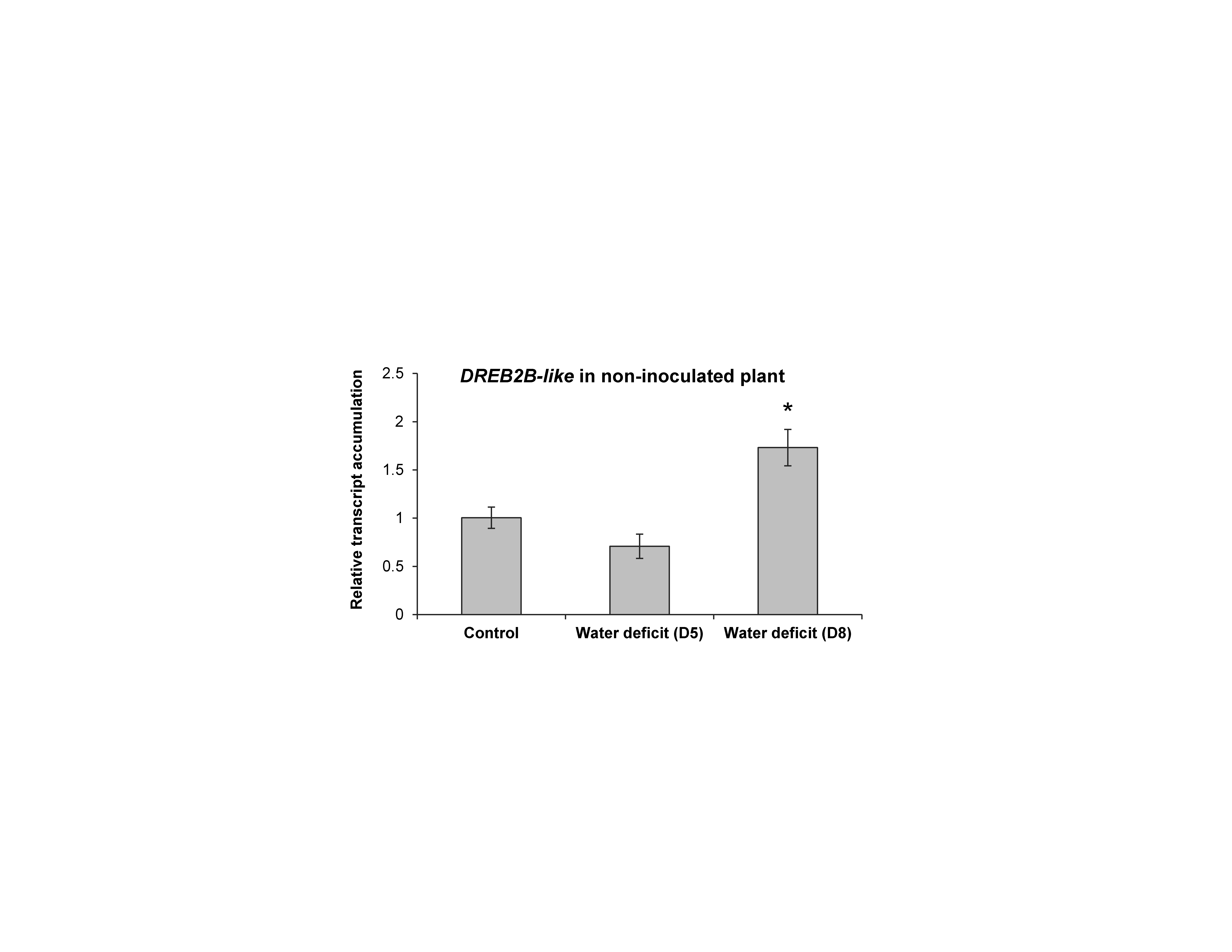

Supplement: S3 Fig — Relative mRNA abundance of Dehydration-Responsive Element-Binding protein 2B-like (DREB2B-like) in non-inoculated plants before after 5 and 8 days of chronic drought stress. * Represents a statistically significant difference. (TIFF) [file pone.0130456.s003.tiff]
