# Supplementary material for: Accelerated Growth Rate and Increased Drought Stress Resilience of the Model Grass Brachypodium distachyon Colonized by Bacillus subtilis B26
Source: PLoS One. 2015 Jun 23;10(6):e0130456. doi: 10.1371/journal.pone.0130456 (PMC4477885; doi:10.1371/journal.pone.0130456)
Supplement: S1 Table — (DOCX) [file pone.0130456.s004.docx]

**S1 Table.** List of specific and universal primers used in quantitative PCR assays.

| **Function** | **Target gene** | **Forward and reverse primer sequences** | **Primer Tm** | **Amplicon size (bp)** | **GenBank for target gene** | **Query/ Reference** |
| --- | --- | --- | --- | --- | --- | --- |
| Drought-responsive | *DHN3-like* | CTCCAGCTCGTCCGAGGAT | 58.8 | 112 | XM_003574949.1 | ABO14458.1 |
|  |  | AGCCATGTGCTGCTGGTTAT | 57.2 |  |  |  |
|  |  |  |  |  |  |  |
|  | *LEA14A-like* | TCGACTACGAGATGCGGGTC | 58.7 | 115 | XM_003565767.1 | NP_171654 |
|  |  | CAGAAGATGTCGGAGAGCGTG | 57.6 |  |  |  |
|  |  |  |  |  |  |  |
|  | *DREB2B-like* | AGCTGACGACCTCTTTGAGC | 57.2 | 110 | XM_003568607.1 | BAA36706 |
|  |  | CTACCGGGTCAGCTTCCATC | 57.4 |  | XM_003568608.1 |  |
|  |  |  |  |  |  |  |
| Methyltransferases | *MET1B-like* | AGACCTCCCACCTCTCTTGG | 58.2 | 101 | XM_003561293.1 | NP_199727.1 |
|  |  | GCTCAGTCTCCAATTGGCCT | 57.5 |  |  |  |
|  |  |  |  |  |  |  |
|  | *CMT3-like* | GATCGCGTGCAACAGATTCC | 56.8 | 110 | XM_003571630.1 | NP_177135.1 |
|  |  | ACTCGCTGAACTTCTGGGTC | 56.9 |  |  |  |
|  |  |  |  |  |  |  |
|  | *DRM2-like* | AAGAAGACAGCTCAACTGCGTGC | 60.0 | 77 | XM_003575408.1 | NP_196966.2 |
|  |  | TTGCAAGAGCACATTGGATCCGC | 60.5 |  |  |  |
|  |  |  |  |  |  |  |
| Internal Standard | *Bradi18S* | GAAGTTTGAGGCAATAACAGGTCT | 55.3 | 131 | XM_003579769.1 | [65] |
|  |  | ATCACGATGAATTTCCCAAGATTAC | 53.5 |  |  |  |
|  |  |  |  |  |  |  |
|  | *SamDC* | AGCGAGTCGACGATACCCTT | 57.9 | 190 | DV482676 | [66] |
|  |  | TGCTAATCTGCTCCAATGGC | 55.4 |  |  |  |
|  |  |  |  |  |  |  |
| Quantification | *Bacillus subtilis* B26 | CAAGTGCCGTTCAAATAG | 48.7 | 565 | JN_689339 | [19] |
|  |  | CTCTAGGATTGTCAGAGG | 48.3 |  |  |  |
|  |  |  |  |  |  |  |
