# Supplementary material for: Accelerated Growth Rate and Increased Drought Stress Resilience of the Model Grass Brachypodium distachyon Colonized by Bacillus subtilis B26
Source: PLoS One. 2015 Jun 23;10(6):e0130456. doi: 10.1371/journal.pone.0130456 (PMC4477885; doi:10.1371/journal.pone.0130456)
Supplement: S2 Table — (DOCX) [file pone.0130456.s005.docx]

**S2 Table**. Nutrient analysis of above ground of control (C) and inoculated *Brachypodium*

with *B. subtilis* 26 (B+).

| **Concentration of Nutrients of Above Ground Tissues (mg/g)*** | | | | | |
| --- | --- | --- | --- | --- | --- |
| **Treatment** | **Days post inoculation (dpi)** | **Nitrogen (N)** | **Phosphorus (P)** | **Potassium (K)** | **Magnesium (Mg)** |
| B+ | 28 | 32.83a | 7.98a | 29.47a | 1.59a |
| Control | 28 | 38.38a | 7.60a | 3.07a | 1.75a |
| B+ | 42 | 15.75b | 4.39b | 16.39b | 0.85b |
| Control | 42 | 21.52a | 6.55a | 21.92a | 1.11a |

*Tissues were harvested 28 and 42 days post inoculation (dpi) with *B. subtilis*. Analysis data were subjected to one-way ANOVA. The significance of the effect of the treatments was determined via Tukey HSD with a magnitude of the F-value (P ­< 0.05). Treatments were tested in pairwise comparison for each time point dpi
